# The Dynamics of Platelet Activation during the Progression of Streptococcal Sepsis

**DOI:** 10.1371/journal.pone.0163531

**Published:** 2016-09-22

**Authors:** Sinead M. Hurley, Nataliya Lutay, Bo Holmqvist, Oonagh Shannon

**Affiliations:** 1 Division of Infection Medicine, Department of Clinical Sciences, Lund University, Lund, SE- 22184 Lund, Sweden; 2 Imagene-iT AB, Medicon Village, SE22381 Lund, Sweden; Royal College of Surgeons in Ireland, IRELAND

## Abstract

Platelets contribute to inflammation however, the role of platelet activation during the pathophysiological response to invasive bacterial infection and sepsis is not clear. Herein, we have investigated platelet activation in a mouse model of invasive *Streptococcus pyogenes* infection at 5, 12, and 18 hours post infection and correlated this to parameters of infection. The platelet population in *ex-vivo* blood samples showed no increased integrin activation or surface presentation of CD62P, however platelet-neutrophil complex formation and plasma levels of CD62P were increased during bacterial dissemination and the progression of sepsis, indicating that platelet activation had occurred *in vivo*. Platelet-neutrophil complex formation was the most discriminatory marker of platelet activation. Platelet-neutrophil complexes were increased above baseline levels during early sepsis but decreased to significantly lower levels than baseline during late sepsis. The removal of these complexes from the circulation coincided with a significant increase in organ damage and the accumulation of platelets in the liver sinusoids, suggesting that platelet activation in the circulation precedes accumulation of platelets in damaged organs. The results demonstrate that monitoring platelet activation using complementary methods may provide prognostic information during the pathogenesis of invasive *S*. *pyogenes* infection.

## Introduction

When bacteria invade the bloodstream the diverse antibacterial mechanisms of the immune response are rapidly mobilized. In some cases an initial controlled host response becomes pathologically augmented and deregulated resulting in sepsis, which may escalate to septic shock with high mortality [1]. Platelets are significant sentinel cells of the blood and are primed to respond to vascular dysfunction and maintain haemostasis. The combined effects of the pro-inflammatory immune response and endothelial dysfunction leads to activation of the coagulation system in sepsis [2]. Activation of the coagulation system may lead to platelet activation and consumption of platelets during sepsis, however the events involved are not clear. Thrombocytopenia occurs during sepsis and has been reported to correlate with the severity of disease [3–5]. Thrombocytopenia appears to be a late stage event in sepsis and may be an independent predictor of organ damage and mortality in critically ill patients [6]. The decrease in platelet count may be a secondary result of uncontrolled plasma coagulation or may be preceded by direct platelet activation during acute inflammation. Studies of platelet activation in sepsis patients have reported both increased [7–9], but also decreased platelet activation [10]. Furthermore, some invasive bacterial pathogens have been reported to directly mediate platelet activation *in vitro* in healthy donors [11], and *ex vivo* in blood from patients infected with the same bacteria [12]. The contradictory results reported for platelet activation in sepsis patients likely reflect the heterogeneity of this patient population both with regard to underlying pathogen and stage of infection, combined with the use of different assays of platelet function in individual studies. *Streptococcus pyogenes* is an important human pathogen that has been ranked as the ninth leading cause of death due to infection globally, mainly due to invasive infection and rheumatic heart disease [13]. The incidence of invasive *S*. *pyogenes* infection, such as streptococcal toxic shock syndrome (STSS), varies significantly geographically and is associated with a particularly high mortality rate [14]. *S*. *pyogenes* AP1 can directly mediate rapid and potent activation and aggregation of human platelets in vitro [15], [16]. We have previously demonstrated that platelets contribute to bacterial dissemination in a mouse model of invasive *S*. *pyogenes* AP1 infection and platelet activation was observed [17]. The aim of present study was to investigate the kinetics of platelet activation during the progression of streptococcal sepsis, using complementary assays of platelet function, and correlate this to the severity of infection.

## Materials and Methods

### Animals

Female Balb/c mice were purchased from Charles River Laboratories, Germany. These mice have previously been demonstrated to be susceptible to invasive infection with *S*. *pyogenes* AP1 infection [18], [19], [17]. The animals were housed under standard conditions of light and temperature, and fed laboratory chow and water *ad libitum*. Experiments were carried out when the mice were 9–13 weeks old.

### Ethics statement

All animal experiments were approved by the local ethics committee at Lund University, Lund, Sweden.

### Animal model of *S*. *pyogenes* infection

*S*. *pyogenes* AP1 is a clinical isolate of the *emm1* serotype (40/58 strain from the World Health Organisation Collaborating Centre for References and Research on Streptococci, Institute of Hygiene and Epidemiology, Prague, Czech Republic). The bacteria were grown in Todd Hewitt broth at 37°C in the presence of 5% CO_2_. Cells were harvested at OD_620_ = 0.5 and washed in PBS. Bacteria (2 x 10^7^ CFU per animal) were administered by intraperitoneal injection. The bacteria dose administrated was verified by viable count determination of the bacterial solution after injection.

Animals were sacrificed by inhalation of CO_2_ at 5, 12 or 18 hours after initiation of infection. These time points were chosen to reflect a late, middle and early stage of infection. We have previously demonstrated that Balb/c female mice succumb to infection within 20 to 24 hours of administration of the same dose of *S*. *pyogenes* [18], therefore 18 hours is a late stage of infection at which animals survive. No animals died without euthanasia in the present study. At all time points a citrated blood sample was obtained by cardiac puncture, the spleen, and liver were removed and bacterial load was determined. The organs were homogenised in 5 ml sterile D-PBS and serial dilutions were plated on Todd Hewitt agar plates for viable count determination after overnight incubation at 37°C in the presence of 5% CO_2_. Prior to homogenisation the intact organ was weighed and the bacterial count was determined relative to the weight of the liver or spleen.

### Flow cytometry

Citrated whole blood samples were obtained by cardiac puncture. Flow cytometry was used to determine the platelet count, neutrophil count, and the activation status of these cells. 20 μl of whole blood was mixed with 40 μl of HEPES buffer pH 7.4 and treated with buffer (unactivated), fMLF (1 μM), or Thrombin (1U/ml) for 5 minutes as positive controls for neutrophil activation or platelet activation, respectively. Five μl of each antibody within the neutrophil or platelet panel was added and incubated for 10 minutes at room temperature prior to red cell lysis and fixation with the Uti-lyse kit (Dako Cytomation) within 30 to 60 minutes of animal sacrifice. Samples were analysed using a BD Accuri C6 flow cytometer. For neutrophil studies, the neutrophil populations were identified as Ly6gPE positive events (BD Biosciences) and platelet-neutrophil complexes within this population were identified as CD41FITC positive events (BD Biosciences). The activation status of neutrophils was determined as up-regulation of CD11b (CD11b PE-Cy5, BD Biosciences). For platelet studies, separate samples were analysed using logarithmic settings to focus on the platelet population of CD41eFluor positive events (eBiosciences, and activated platelets within this population were determined as CD62P FITC or JONA.PE (Emfret Technologies) positive events.

### Determination of plasma CD62P and AST

Plasma was prepared by centrifugation of citrated blood samples at 2000g for 15 minutes and stored at -80°C until analysis. The soluble CD62P levels in plasma were determined using an ELISA kit (Abcam), according to the manufacturers’ instructions. AST was determined using an activity kit according to the manufacturers’ instructions (Sigma Aldrich).

### Immnohistochemistry and immunofluorescent labelling of organs

Paraffin-embedded lung and liver tissues were sectioned at a thickness of 5 μm and mounted on SuperFrost^®^ Plus glass slides (Menzel-Gläser, Germany). The slides were de-paraffinized and re-hydrated followed by blocking of endogenous peroxidase activity by application of hydrogen peroxide for 10 min. After blocking with 5% goat serum and 1% BSA in PBS containing 0.1% Triton X-100, for 1 h at room temperature (RT), sections were incubated with rabbit polyclonal antibodies against mouse CD41 (ab63983, Abcam) overnight at 4°C. Primary antibody binding sites were visualized using DAKO EnVision+ System-HRP, performed according to the manufacturers’ instructions. For counterstaining, nuclei were labeled with Mayer's hematoxylin (Histolab, Gothenburg, Sweden). Sections were then dehydrated, mounted and cover slipped in Pertex (Mayers, Histolab). Analyses were performed with a bright-field microscope (Olympus IX73).

Simultaneous visualization of platelets and neutrophils was performed via immunofluorescence double labelling. Neutrophils were visualized using rat monoclonal antibodies against mouse Ly6g (ab63983, Abcam) and platelets using rabbit polyclonal antibodies against mouse CD41 (ab63983, Abcam). Nonspecific antibody binding sites were blocked with 5% goat serum and 1% BSA in PBS containing 0.1% Triton X-100, for 1 h at RT. Incubations with a cocktail of anti-Ly6g and anti-CD41 were performed overnight at 4°C. After rinses in PBS, sections were incubated for 1 h at RT with a cocktail of secondary antibodies, comprising Rhodamine Red-conjugated donkey anti-rat IgG (H+L) and Alexa Fluor^®^ 488-conjugated donkey anti-rabbit IgG (H+L) (712-296-153 and 711-546-152, Jackson ImmunoResearch). Following rinses in PBS, cell nuclei were stained with 4',6-diamidino-2-phenylindole (DAPI) for 30 min at RT. Sections were then mounted in Fluoroshield Mounting (ab104135, Abcam,) and cover slipped. Analyses of the double labelled sections were performed using an epifluorescence microscope (Olympus, IX73) and confocal microscopy using a Zeiss confocal laser-scanning microscope (CLSM, LSM 510 META).

### Statistical methods

Statistical analysis was performed using Prism (GraphPad Software). The students t-test was used to investigate differences when the data were normally distributed or otherwise with a Mann-Whitney U test. All p-values are uncorrected for multiple comparisons. Correlation was assessed with Spearman’s rank correlation test.

## Results

### The kinetics of weight loss and bacterial dissemination in *S*. *pyogenes* infected animals

**Fig 1:**
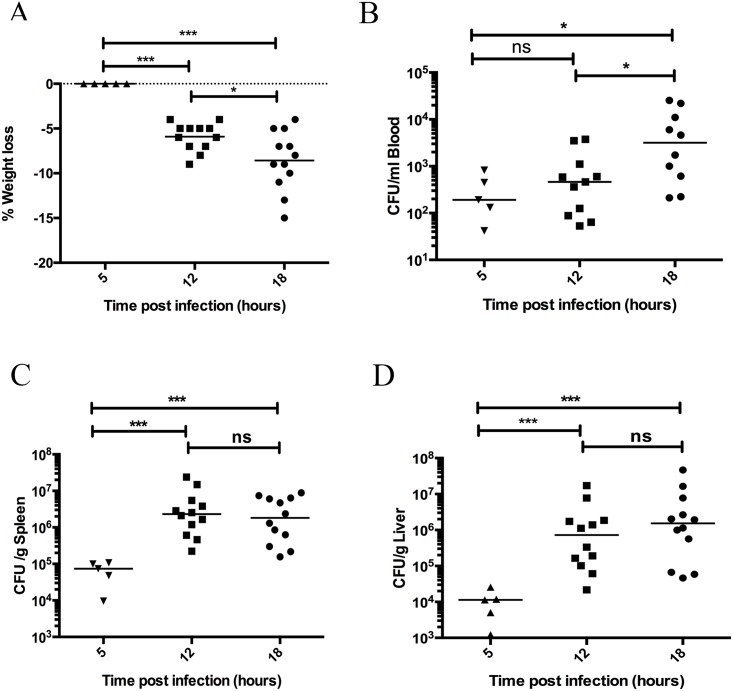
Weight loss and bacterial dissemination increases over time during invasive infection. Mice infected with *S*. *pyogenes* AP1 were weighed before infection and at 5, 12, and 18 hours post infection and the decrease in body weight was calculated (A). At 0, 5, 12, and 18 hours post infection the mice were sacrificed the bacterial load in blood (B), spleen (C), and liver (D) was determined by serial dilution of homogenates and viable count determination. Horizontal line (A) represents the mean, p-value calculated using a t-test. Horizontal line (B-D) represents median, p-value calculated using a Mann-Whitney U test.

During systemic murine infection animals decrease in body weight and this is a sensitive marker of the overall health status of these animals [20]. Body weight was determined prior to infection and at 5, 12, and 18 hours post infection, and the percentage weight loss over time was calculated. As expected no significant weight loss occurred early during the infection, within 5 hours (Fig 1A). Weight loss was significantly increased after 12 (p = 0.0002) and 18 hours (p = 0.0002) and between 12 and 18 hours there was a significant increase in weight loss (p = 0.03) (Fig 1A). This demonstrates a progressive deterioration in the health status of the animals during the progression of the infection. Bacterial dissemination from the local peritoneal site of administration through the bloodstream to distant organs was also determined. In a pilot experiment bacterial load to kidney, lung, liver, and spleen was observed. Kidney and spleen gave equivalent bacterial loads, liver was slightly lower and lung was lowest or not infected. Quantitative and comparative analyses were therefore only performed for liver and spleen samples. Bacteria were present in the bloodstream within 5 hours however, no significant increase occurred at 12 hours, and significantly increased bacteria load was only observed at 18 hours post infection (p = 0.013) (Fig 1B). Bacteria were present in the organs within 5 hours and the bacteria load was significantly increased in spleen and liver at 12 (p = 0.0002 and p = 0.0003, respectively) and 18 hours (p = 0.0003 and p = 0.0006, respectively) post infection, however no further increase was observed between 12 and 18 hours (Fig 1C & 1D). This demonstrates that the bacteria load in the organs stabilises between 12 and 18 hours, while the bacteria load in the blood increases and this may reflect the ability of *S*. *pyogenes* to survive in blood.

### Neutrophils are mobilised and platelets are consumed in response to infection

**Fig 2:**
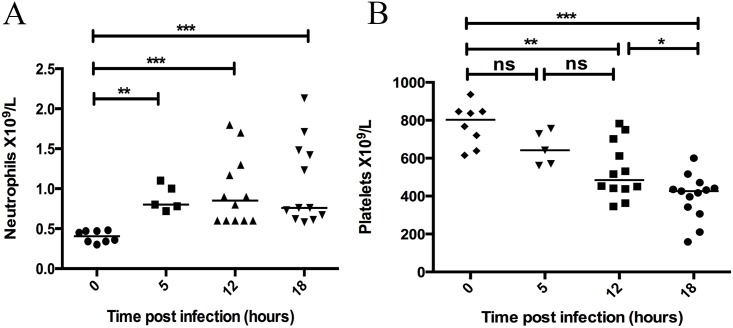
Circulating neutrophil counts increase rapidly during invasive infection, while platelet counts decrease progressively over time. At 0, 5, 12, and 18 hours post infection with *S*. *pyogenes* the mice were sacrificed and a blood sample was taken by cardiac puncture. Flow cytometry of citrated blood was used to determine neutrophil (A) and platelet counts (B) in each animal. Horizontal line represents median, p-value calculated using a Mann-Whitney U test.

Neutrophils and platelets were counted in blood samples using flow cytometry. The total neutrophil count in normal healthy mice was relatively low and homogeneous and within 5 hours of infection there was a significant increase in neutrophil counts (p = 0.0016), which continued to increase at 12 (p = 0.0003), and 18 hours (p = 0.0001) post infection (Fig 2A). At 5 hours post infection the circulating platelet count began to decrease but was not significantly affected (Fig 2B). At 12 (p = 0.0015) and 18 hours (p = 0.0001) infected animals exhibited significantly decreased platelet numbers, and from 12 to 18 hours the platelet counts were further depleted (p = 0.015) (Fig 2B). Collectively, the data demonstrate that both neutrophils and platelets rapidly respond to the infection, however the platelet count more distinctly differentiates between early, middle and late infection time points.

### Platelet activation occurs during invasive infection, however the results are highly assay dependent

**Fig 3:**
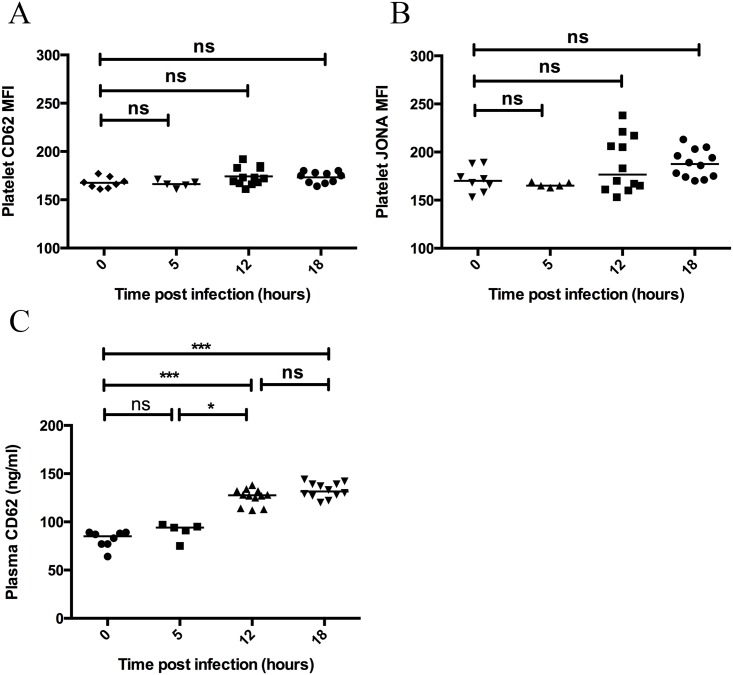
Activated platelets are not found in ex-vivo blood samples during invasive infection however plasma levels of released CD62P are significantly increased over time. At 0, 5, 12, and 18 hours post infection with *S*. *pyogenes* the mice were sacrificed and a blood sample was taken by cardiac puncture. Flow cytometry of citrated blood was used to determine the activation status of the circulating platelet population activated platelets as median of the fluorescence (MFI) for CD62P (A) and JONA (B). Plasma was prepared and soluble CD62P was determined by ELISA (C). Horizontal line represents median, p-value calculated using a Mann-Whitney U test.

The occurrence of thrombocytopenia suggests that circulating platelets were consumed during the acute infection. We aimed to determine if platelet activation could be detected in association with this depletion of circulating platelets. Activation of platelets was determined by flow cytometry for the presentation of CD62P (Fig 3A) and activation of the GPIIb/IIIa integrin on the platelet surface (Fig 3B). No significant increase was observed at any time point (Fig 3A & 3B) indicating that the platelet population in the *ex-vivo* sample were not activated. This was not due to an inability to become activated since platelet activation occurred on addition of thrombin to the *ex-vivo* blood samples (data not shown). Activated platelets may preferentially adhere to one another and the endothelium, or accumulate in small vessels, and are therefore difficult to obtain in an *ex vivo* blood sample. A tendency towards increased activation of the GPIIb/IIIa integrin on the platelet surface was observed at 12 and 18 hours post infection (Fig 3B). This suggests that integrin activation may be a more sensitive marker of in-vivo platelet activation than surface CD62P, which is shed from activated platelets [21]. The inability to detect platelet bound CD62P may also reflect shedding of this molecule from the platelet surface during sample preparation. In order to further investigate whether platelet activation had occurred *in vivo*, we therefore investigated CD62P levels in plasma. Importantly, plasma CD62P levels were significantly increased at 12 (p = 0.0001) and 18 hours (p = 0.0001) post infection, however there was no significant difference from 12 to 18 hours (Fig 3C). This suggests that platelet activation had occurred *in vivo* in response to infection.

### Platelet-Neutrophil complex formation is initially increased during invasive infection and decreases at a late time point

**Fig 4:**
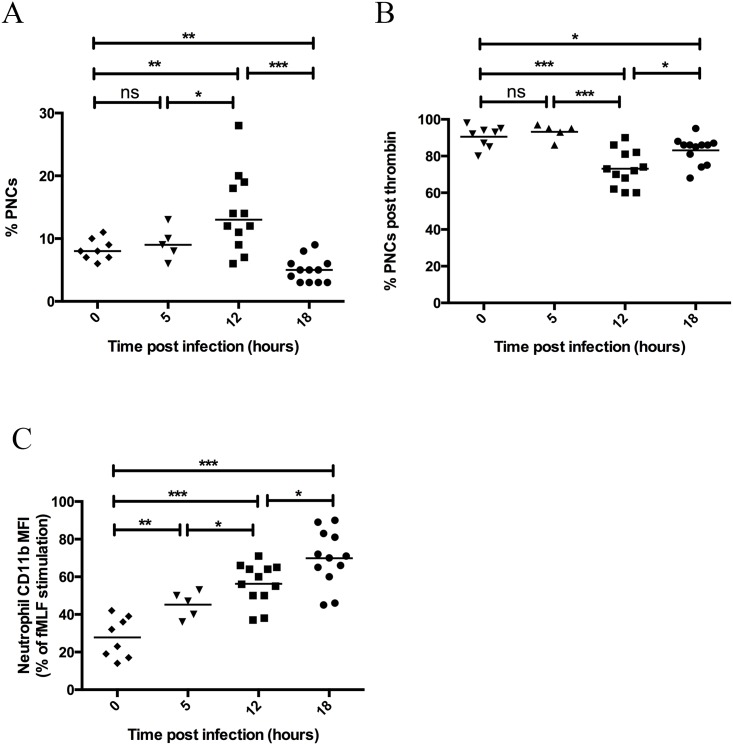
Platelet-Neutrophil complex formation is increased during the early stages of invasive infection and decreased during late stage infection. At 0, 5, 12, and 18 hours post infection with *S*. *pyogenes* the mice were sacrificed and a blood sample was taken by cardiac puncture. Flow cytometry of citrated blood was used to determine formation of platelet-neutrophil complexes in vivo (A) or post addition of thrombin ex vivo (B). Upregulation of CD11b on neutrophils was also determined (C). The median of the fluorescence for CD11b was measured for each sample with and without prior stimulation with fMLF ex-vivo. Stimulation with fMLF for each sample was set to 100% and the values displayed are for unstimulated samples. Horizontal line represents mean, p-value calculated using a t-test with Welch’s correction for unequal variances.

In response to activation, platelets adhere to neutrophils to form heterogeneous complexes with one another that is dependent on platelet activation [22], [23]. Platelet-neutrophil complex (PNC) formation was determined by flow cytometry of platelet events within the neutrophil population. PNC formation was significantly increased at 12 hours post infection (p = 0.0079), however complex formation was significantly decreased from normal levels at 18 hours post infection (p = 0.0011) (Fig 4A). The neutrophil count was increased at both 12 and 18 hours, demonstrating that the decrease in complex formation did not reflect a diminished availability of neutrophils. The platelet count was decreased at 12 and 18 hours (Fig 2B) but the circulating platelet numbers were still higher than neutrophils and the possibility remains to form PNCs. This was confirmed by our findings that PNCs could be formed on addition of thrombin to the *ex-vivo* blood samples (Fig 4B). There was however a significantly decreased level of PNC formation in response to thrombin in the samples taken after 12 (p = 0.0001) and 18 hours (p = 0.011), which likely reflects the high level of PNC formation that had already occurred in these populations (Fig 4A & 4B). We therefore conclude that PNCs occur during the infection, but at later time points these PNCs are no longer detected in the circulating blood and may be sequestered in the vessels.

As expected, neutrophils became activated in response to infection and significantly increased CD11b presentation at the neutrophil surface at 5 hours (p = 0.0048), 12 hours (p = 0.0001), and 18 hours (p = 0.0001) post infection (Fig 4C). Neutrophil activation increased early and over time while PNC formation first increases after 12 hours and then decreases during the progression of infection, indicating that the neutrophil activation and PNC formation are not correlated to one another. This indicates that PNC formation is dependent on platelet activation.

### Platelet aggregates accumulate in liver sinusoids as the infection progresses

**Fig 5:**
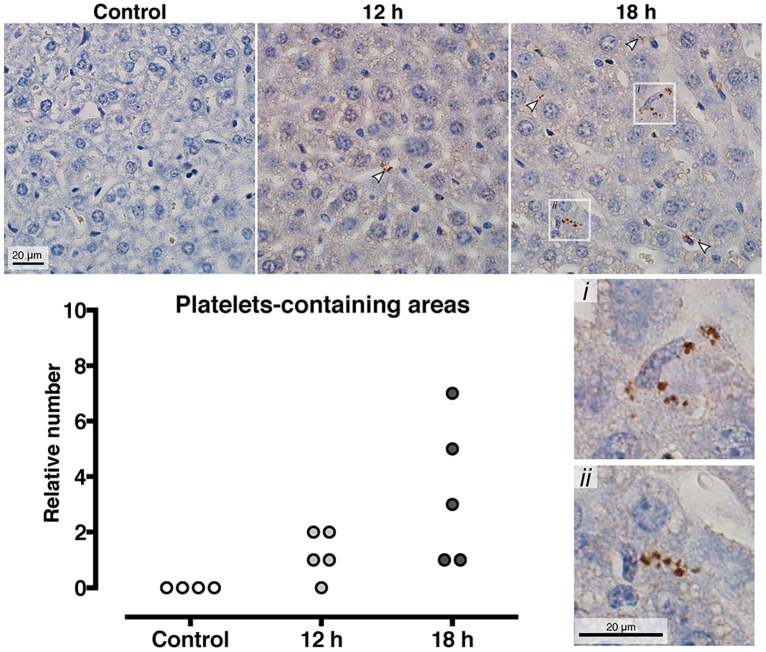
Platelets are progressively accumulated in the liver sinusoids at during invasive infection. At 0, 12, and 18 hours post *S*. *pyogenes* infection the mice were sacrificed and the liver was harvested, fixed and embedded in paraffin blocks, sectioned and immunohistochemistry was performed with anti CD41 to detect platelets. Representative images are shown and quantification of the number of platelet aggregates per slide is illustrated as a column graph, where each dot is an individual mouse.

In an initial experiment thin sections of lung and liver tissue from three mice obtained at the late stage of infection (18 hours post infection) were stained for neutrophil and platelet infiltration. Neutrophils were observed in both the liver and the lung, however no platelets were detected in the lung using our experimental system (data not shown). Comparative quantitative immunohistochemistry of platelet infiltration over time was therefore subsequently performed on liver tissue from individual mice prior to infection (n = 3), 12 hours post infection (n = 5), and 18 hours post infection (n = 5). Platelets were not observed in the livers of the healthy mice, and platelets were only observed in infected animals. The platelets occurred in aggregates sporadically throughout the tissue but were mainly found in aggregates within the liver sinusoids (representative images in Fig 5). The number of platelet positive areas observed per liver section was recorded. No platelet containing areas were detected in healthy animals, 1–2 areas were observed in 4/5 mice after 12 hours of infection and this increased to 2–8 areas in 5/5 mice at 18 hours post infection (Fig 5). Immunofluorescence double labelling of the liver sections demonstrated platelet aggregates that contained small DAPI positive elements, with a size and morphology corresponding to bacterial cocci. Since platelets do not contain DNA and are DAPI negative, this implies that the platelets may co-localise with *S*. *pyogenes* in the infected organs (S1 Fig). No co-localisation of platelets and neutrophils was observed in the infected liver sections (S1 Fig).

### Organ damage occurs late during streptococcal sepsis and is correlated to platelet activation and thrombocytopenia

**Fig 6:**
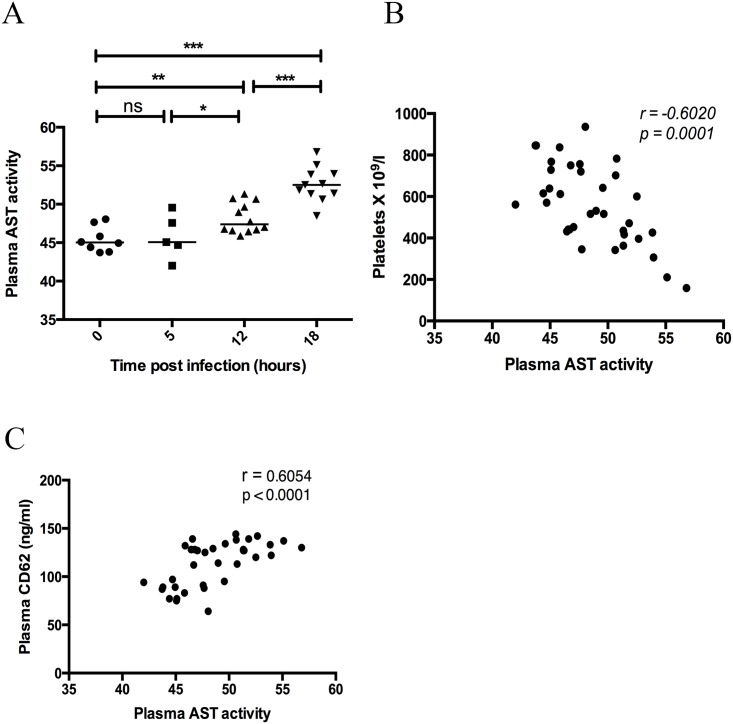
Organ damage increases over time during invasive infection and correlates with platelet activation and thrombocytopenia. At 0, 5, 12 and 18 hours post *S*. *pyogenes* infection the mice were sacrificed and a blood sample was taken by cardiac puncture. Plasma was prepared and AST activity was determined using a commercial kit (A). Horizontal line represents median, p-value calculated using a Mann-Whitney U test. Correlation between plasma AST activity and platelet count (B) or plasma AST activity and plasma CD62 levels (C) was performed using a Spearman’s rank correlation.

Aspartate Aminotransferase (AST) is released from damaged cells, including liver, heart, kidney or muscle cells. Plasma levels of AST were determined in order to assess organ damage in infected animals over the time course of the infection. AST activity in plasma was significantly increased at both 12 hours (p = 0.005) and 18 hours (p = 0.0001) and the levels continued to increase from 12 to 18 hours (p = 0.0001). This is the same time point at which a significantly higher number of platelet aggregates were observed in the liver, and we propose that activated platelets have accumulated in infected and damaged organs. This is supported by the observation that the level of organ damage (AST activity) correlated with the increase in a plasma marker of platelet activation (Fig 6C) and the decrease in circulating platelets, thrombocytopenia (Fig 6B). The results suggest that platelet activation precedes the appearance of platelet aggregates in damaged organs and the removal of platelets from the circulating pool.

## Discussion

Platelets patrol the circulation and rapidly become activated in response to endothelial dysfunction, activation of coagulation factors and pro-inflammatory mediators. During invasive streptococcal infection all of these agonists may become available and platelet activation may occur. *S*. *pyogenes* does not mediate direct activation of mouse platelets in-vitro, since mouse platelets lack the Fc receptor required for activation of human platelets. Herein we investigated multiple parameters of platelet activation during streptococcal sepsis at an early (5 hours), middle (12 hours) and late phase (18 hours), as the infection progresses and animal health deteriorates.

We report that platelet activation does occur during streptococcal sepsis. The response was highly dependent on the choice of the platelet activation assay; platelet-neutrophil complex formation occurred and plasma CD62P levels were increased. Endothelial cells can also release CD62P, however platelets are reported to be the predominant source of plasma CD62P and this is a robust marker of platelet activation [24] [25]. PNC formation is dependent on platelet activation and upregulation of CD62P to the platelet surface [23]. Platelets have been demonstrated to participate in innate immune responses in mouse models of LPS induced sepsis [26], [27], however upregulation of CD62P to the platelet surface was not elevated in these studies. Importantly, formation of platelet-neutrophil complex (PNCs) has been demonstrated to be increased in mice post administration of LPS [28]. This underlines in importance of combining platelet activation assays in order to detect in-vivo activation of platelets.

Among the few studies of PNC formation in sepsis patients, PNC formation has been detected although other parameters of platelet activation were not increased [29]. PNC levels have also been reported to be elevated in patients with uncomplicated sepsis, while levels decreased in patients with multiple organ failure [30]. This is in agreement with our findings of increased PNC formation during the middle phase of sepsis and diminished PNC formation during late stage sepsis. Furthermore, we detected increased accumulations of platelet aggregates in the livers of animals at the same time point. This demonstrates that activated platelets adhere to one another and other cells and may become sequestered in the small vessels where they can contribute to diminished perfusion and organ damage.

Intravital microscopy of liver sinusoids directly treated with LPS has demonstrated platelet dependent stimulation of neutrophil extracellular trap formation [28] and local entrapment of bacteria in these aggregates [31]. In our present study we have detected aggregates of platelets and bacteria together in the infected liver, which implies that a direct platelet-bacteria interaction occurs. The interaction between platelets and bacteria may represent a host defence strategy to entrap pathogenic bacteria. Platelet innate immune signalling has previously reported not to contribute to a successful immune response to bacterial sepsis [32], while platelet CD62P was reported to be important for a successful immune response to bacterial sepsis [33]. Alternatively, the activation of platelets during infection may be a bacterial strategy to encloak itself in host platelets and evade immune cells during passage through the circulation. We have previously observed that animals depleted of platelets prior to infection have a significantly decreased bacterial dissemination to distant organs post streptococcal infection, implying that platelets contribute to the bacterial survival during invasive streptococcal infection [17]. Collectively, these findings underline the diversity of the platelet response during sepsis caused by diverse pathogens. We propose that platelet activation and in particular PNC formation may represent an important biomarker for distinction of patients during the progression from early to late stages of sepsis and platelet infiltration may precede organ damage.

## Supporting Information

S1 Fig Co-localisation of platelets with bacteria but not neutrophils in the liver. (PDF)
