# Supplementary material for: The Dynamics of Platelet Activation during the Progression of Streptococcal Sepsis
Source: PLoS One. 2016 Sep 22;11(9):e0163531. doi: 10.1371/journal.pone.0163531 (PMC5033464; doi:10.1371/journal.pone.0163531)
Supplement: S1 Fig — (PDF) [file pone.0163531.s001.pdf]

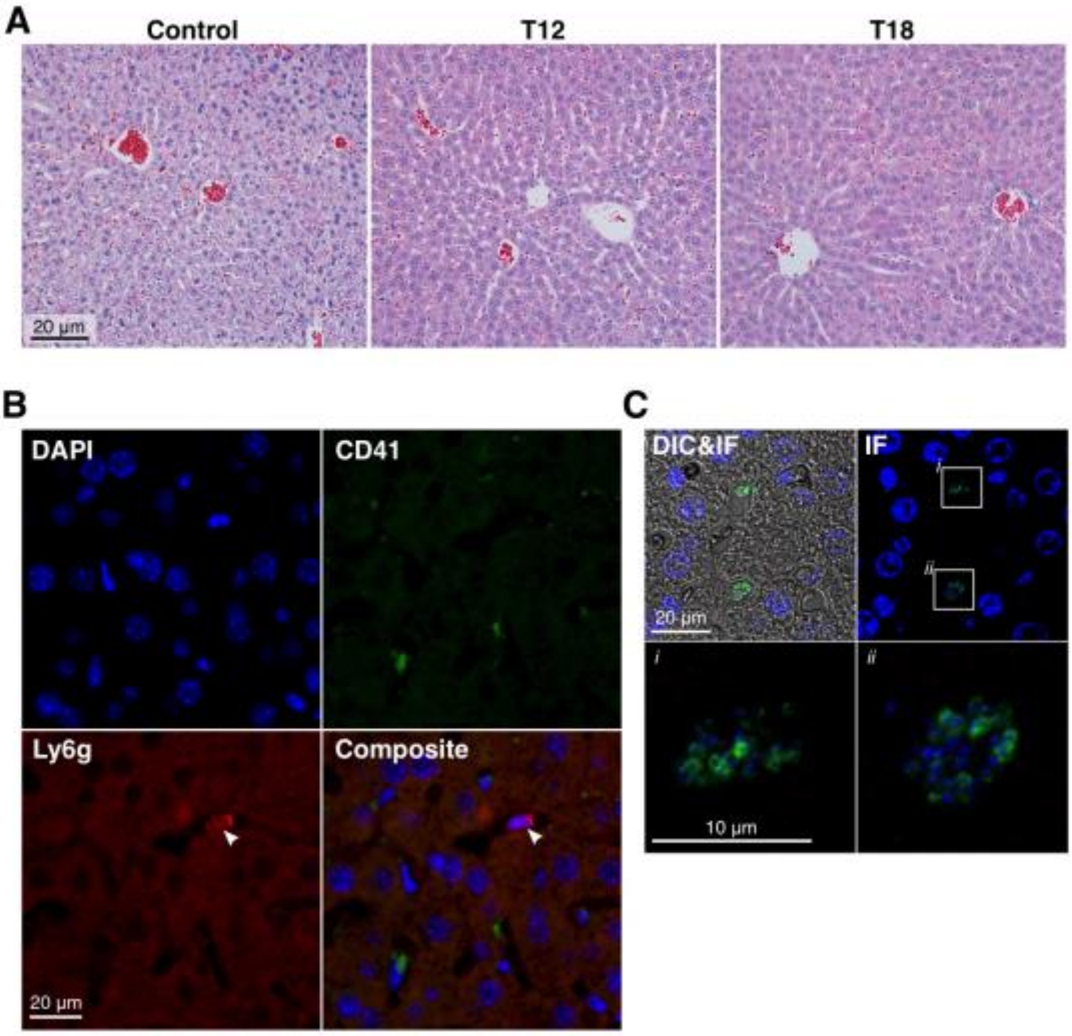

S1 Fig. Co-localisation of platelets with bacteria but not neutrophils in the liver

Haematoxylin and eosin stained sections of livers (uninfected control and infected for 12 and 18 h - T12 and T18 (**A**). Epifluorescence (**B**) and confocal microscopy (**C**) of a liver section 18 h post infection. Double immunolabelling of the liver sinusoids demonstrate platelets (green) and neutrophils (red, shown by an arrowhead). The nuclei of the liver cells were counterstained with DAPI (blue) **(B)**. Confocal differential interference contrast (DIC) and

immunofluorescence (IF) images indicate that platelet aggregates (green) co-localized with bacteria stained with DAPI (blue) **(C)**.
